# The effect of compliance with a perioperative goal-directed therapy protocol on outcomes after high-risk surgery: a before-after study

**DOI:** 10.1007/s10877-020-00585-w

**Published:** 2020-09-12

**Authors:** M. F. Boekel, C. S. Venema, T. Kaufmann, I. C. C. van der Horst, J. J. Vos, T. W. L. Scheeren

**Affiliations:** 1 Department of Anesthesiology, University of Groningen, University Medical Center Groningen, Hanzeplein 1, PO Box 30.001, 9700RB Groningen, The Netherlands; 2 Chair of Department of Intensive Care, Maastricht University Medical Center+, Maastricht University, Maastricht, The Netherlands

**Keywords:** Perioperative goal-directed therapy, High-risk surgery, Before-after study, Protocol compliance

## Abstract

Perioperative goal-directed therapy is considered to improve patient outcomes after high-risk surgery. The association of compliance with perioperative goal-directed therapy protocols and postoperative outcomes is unclear. The purpose of this study is to determine the effect of protocol compliance on postoperative outcomes following high-risk surgery, after implementation of a perioperative goal-directed therapy protocol. Through a before-after study design, patients undergoing elective high-risk surgery before (before-group) and after implementation of a perioperative goal-directed therapy protocol (after-group) were included. Perioperative goal-directed therapy in the after-group consisted of optimized stroke volume variation or stroke volume index and optimized cardiac index. Additionally, the association of protocol compliance with postoperative complications when using perioperative goal-directed therapy was assessed. High protocol compliance was defined as ≥ 85% of the procedure time spent within the individual targets. The difference in complications during the first 30 postoperative days before and after implementation of the protocol was assessed. In the before-group, 214 patients were included and 193 patients in the after-group. The number of complications was higher in the before-group compared to the after-group (n = 414 vs. 282; p = 0.031). In the after-group, patients with high protocol compliance for stroke volume variation or stroke volume index had less complications compared to patients with low protocol compliance for stroke volume variation or stroke volume index (n = 187 vs. 90; p = 0.01). Protocol compliance by the attending clinicians is essential and should be monitored to facilitate an improvement in postoperative outcomes desired by the implementation of perioperative goal-directed therapy protocols.

**Electronic supplementary material:**

The online version of this article (10.1007/s10877-020-00585-w) contains supplementary material, which is available to authorized users.

## **Introduction**

Perioperative goal-directed therapy (pGDT) aims to optimize the patient`s hemodynamic status using interventions to reach predefined target values in the perioperative period [1]. These interventions include administering fluids, inotropes, and vasopressors, and are ultimately directed at improving oxygen delivery to organs. pGDT is considered to reduce postoperative complications and length of stay after high-risk surgery (HRS) [2–5]. Hence, the use of pGDT is recommended by several national guidelines, is part of early recovery after surgery (ERAS) guidelines, and has been included in the European Society of Anaesthesiology guideline for non-cardiac surgery [6–10].

Despite such recommendations, pGDT is not yet routinely implemented in clinical practice [11]. One explanatory factor for this discrepancy might be that clinical heterogeneity among trials troubles a clear interpretation of trials evaluating pGDT protocols [12]. Additionally, the term “goal-directed therapy” is poorly defined and is used to describe different treatment strategies in studies with varying degrees of complexity [13]. Furthermore, the successful implementation of a multimodal protocol does not necessarily translate into improved usage of a protocol [14]. Quality improvement programs allow evaluation of new monitoring and treatment strategies such as pGDT in clinical practice. However, the importance of protocol compliance, i.e., whether the treating clinician applies pGDT and achieves the hemodynamic targets in the individual patient, is unclear, since most studies that have evaluated the effect(s) of pGDT either reported a high overall protocol compliance or did not record compliance at all [15–18].

The lack of evidence on the effective use of pGDT by clinicians may further contribute to the uncertainty as to whether pGDT protocols improve postoperative outcomes in clinical practice. We implemented a quality improvement program in which a pGDT protocol was introduced as an addition to clinical practice for patients undergoing HRS. As we have expressed previously, such a ‘before and after’ model allows evaluating the effectiveness of pGDT under real-life conditions [19]. We hypothesized that high compliance with a pGDT protocol improves postoperative outcomes compared to low compliance. While we assumed that the implementation of a pGDT algorithm would decrease the incidence of postoperative complications in patients undergoing HRS in our hospital, we secondarily assessed whether high pGDT protocol compliance contributed to further reduction in postoperative complications.

## **Methods**

### **Study design and patients**

The Local Research Ethics Committee approved the study and waived the need for consent since the pGDT protocol was considered clinical practice for patients undergoing elective HRS in our hospital. The study, undertaken at an academic teaching hospital was set-up using a before-after design [19]. In the before-group, data were retrospectively collected from patients who underwent HRS before the implementation of the pGDT protocol (August 2013 to February 2015). In the after-group, data were prospectively collected from patients undergoing HRS after the implementation of the pGDT protocol (July 2015 to February 2018). Patients in the after-group were compared to historical matched control patients in the before-group who underwent the same surgical procedure. Five major surgical procedures were included for analyzing the effects of the implementation of pGDT: pylorus-preserving pancreaticoduodenectomy (PPPD), abdominoperineal resection (APR), open abdominal aortic aneurysm repair (open AAA), open esophageal resection, and femoral-popliteal artery repair. Patients younger than 18 years, pregnant patients, (partly) thoracoscopic or laparoscopic procedures, and patients undergoing emergency surgery were excluded from this study. This manuscript adheres to the SQUIRE guidelines [20].

### pGDT protocol and anesthetic management

**Fig. 1:**
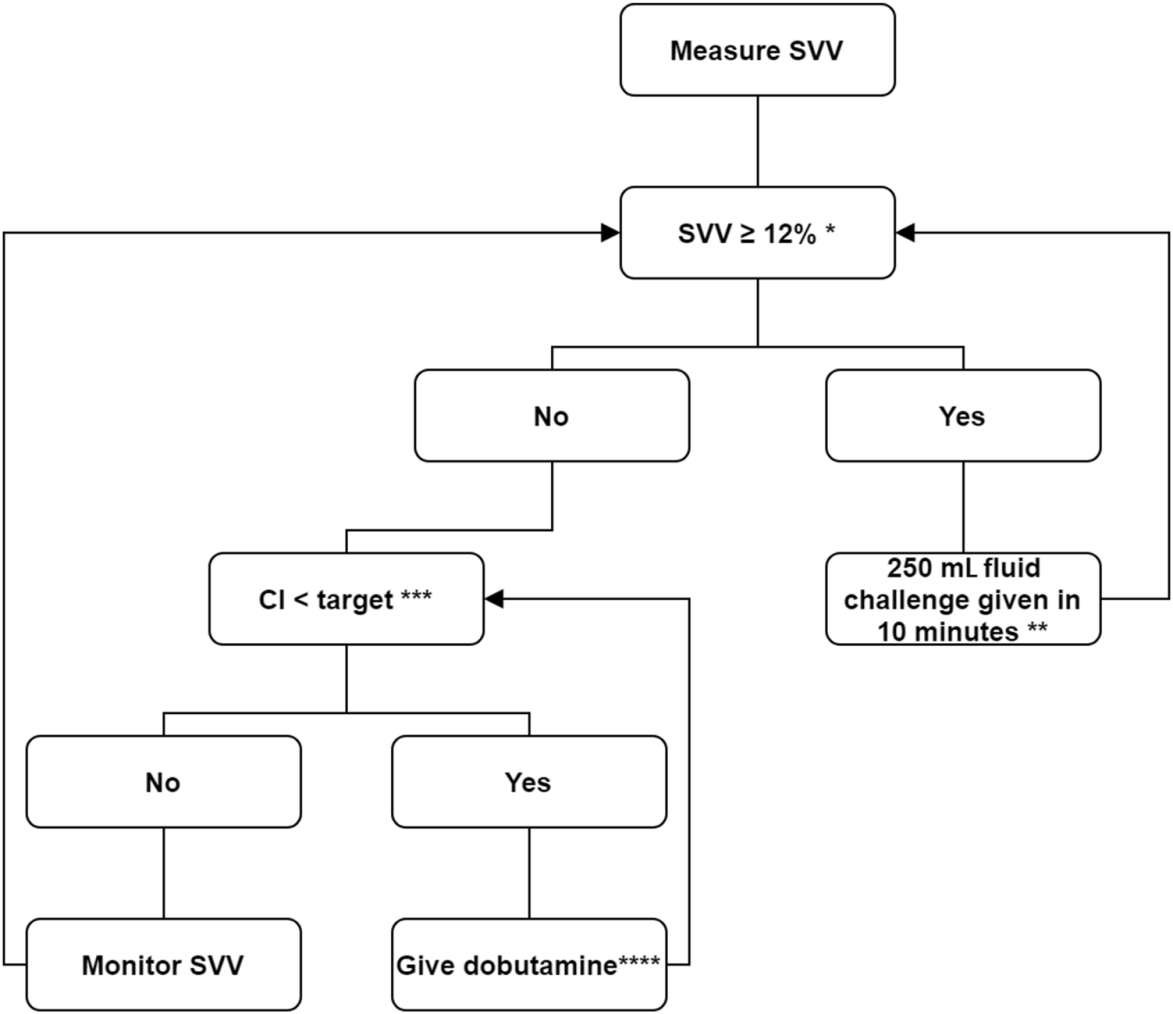
pGDT protocol 1. *SVV* Stroke volume variation, *CI* cardiac index. Both variables were obtained by using the EV1000 monitoring system, utilizing uncalibrated pulse-wave contour analysis The protocol was implemented with the following considerations:

A 4-month training period (January 2015 to May 2015) was used to train caregivers (anesthesiologists and anesthesia nurses) in the pGDT treatment algorithms and associated monitoring methods before implementing pGDT. The training was done by giving lectures and hands-on training—using active learning methods during several internal meetings—on how to optimize the patient`s hemodynamic status using stroke volume variation (SVV), cardiac index (CI), and stroke volume index (SVI). In short, the pGDT protocol consists of two treatment algorithms, based on the applicability of SVV-guided assessment of fluid responsiveness. Applicability of SVV depends on the presence of sinus rhythm, absence of significant valvular heart disease, and the absence of congestive heart failure [21]. The patients’ lungs were mechanically ventilated in a volume-controlled mode with tidal volumes set at ≥ 8 ml kg^− 1^. The SVV-guided treatment algorithm (*pGDT1*; Fig. 1) was primarily based on SVV and cardiac index (CI). The alternative algorithm (*pGDT2*, see Supplementary Figure) was used when SVV guidance was rendered invalid (which, for example, included periods of open chest conditions and one-lung ventilation during esophageal resection) and was primarily based on stroke volume index (SVI) and CI. Both pGDT treatment algorithms propose the use of intravenous fluids (colloids or crystalloids) to improve SVV or SVI, and dobutamine to further augment CI. Caregivers using one of the pGDT treatment algorithms were recommended to improve SVV or SVI *first*, and CI *second*, i.e. after SVV (pGDT treatment algorithm 1)/SVI (pGDT treatment algorithm 2) was within the set target. If the patient was unresponsive to (repeated) fluid bolus administration while the CI target was not reached, the caregiver was recommended to increase CI. The SVI threshold in the 2nd treatment algorithm was set at 35 ml m^− 2^, as based on previous studies [22, 23]. In our institution, the target MAP threshold generally used by caregivers is 65 mmHg, in patients undergoing non-cardiac surgery; nevertheless, it was left at the discretion of the attending anesthesiologist and no further specific recommendations were made in the after group. In all patients, a 20 g radial artery catheter was placed for continuous monitoring of blood pressure. The arterial catheter was connected to the FloTrac/EV1000 monitoring system (Edwards Lifesciences, Irvine CA, USA), which uses uncalibrated pulse-wave analysis to calculate SVI, CI and SVV [24].

As the before-group served as a historical control group for which data were collected retrospectively, hemodynamic management—including the selection, dosing, and timing of fluids, vasopressors, and inotropes, mode of mechanical ventilation—was at the discretion of the attending anesthesiologist.

In both the before-group and the after-group, general anesthesia and/or epidural analgesia was induced and maintained according to institutional practice and was left at the discretion of the attending anesthesiologist. In all patients, depth of anesthesia monitoring was applied using bispectral index monitoring (BIS; Aspect Medical Systems, Norwood, MA, USA) and BIS was targeted between 40 and 60 throughout the procedure.

After the procedure was completed, patients from both groups were transferred to the intensive care unit (ICU) for postoperative care or to the post-anesthetic care unit (PACU) for extended postoperative monitoring. All patients were treated following enhanced recovery guidelines during pre-, per- and postoperative setting if applicable. There were, to the best of our knowledge, no major changes in pre- or post-operative care in any of these protocols during the conduct of the study.

### Data collection

All pre-, intra- and postoperative data were gathered using our electronic medical record database, retrospectively in the before-group and prospectively in the after-group. The collected data included preoperative information such as age, gender, height, weight and procedure type, as well as all relevant continuous intraoperative data from the anesthesia monitor and ventilator, together with data on the use of intravenous fluids and medication (anesthetic agents, vasopressors, inotropes), and the procedure duration, amount of intraoperative blood loss and urine production. Data from the FloTrac/EV1000 monitor were separately collected and after careful synchronization, added to the study database. Postoperative complications during the first 30 postoperative days were also gathered from the electronic patient data management system and classified based on the ‘Expanded Accordion Severity Classification Model’ (see later).

### **Outcomes** 

The primary outcome of this before-after study was the effect of pGDT protocol compliance on the incidence of postoperative complications. Postoperative complications were graded according to the ‘Expanded Accordion Severity Classification Model’, which has six grades of severity: mild, moderate, severe complications without need for general anesthesia, severe complications with a need for general anesthesia, organ failure and postoperative death within 60 days after the procedure [25]. The severity of the complications was given using points ranging from one point for mild complications to six points for postoperative death. The cumulative score was used to quantify postoperative complications.

Protocol compliance was determined by analyzing the total time SVV, SVI and CI were within the predefined target of the two pGDT treatment algorithms. SVV/SVI and CI were analyzed separately using time-weighted averages. High protocol compliance with SVV/SVI or with CI was defined as ≥85%, based on the assumption that the caregiver is familiar with the pGDT protocol: this cut-off point takes into account sudden changes in hemodynamic stability that are inherent to HRS under routine clinical circumstances.

### **Statistical analysis**

SPSS 23.0 for Windows (IBM SPSS Statistics, IBM Corporation, Armonk, NY) was used for the statistical analyses. Descriptive analyses were performed for all variables. Continuous variables are reported as mean with standard deviation for parametric data or median with interquartile range for non-parametric data. Categorical data are reported as numbers (with percentages). An independent sample t-test was used to analyze the parametric data and the Mann-Whitney U test was used for non-parametric data. Chi-square or Fisher’s exact test was performed to analyze categorical data. P-values below 0.05 were considered statistically significant, while the Bonferroni correction was used to reduce the chance of a type 1 error due to multiple testing.

## Results

### **Participants**

**Fig. 2:**
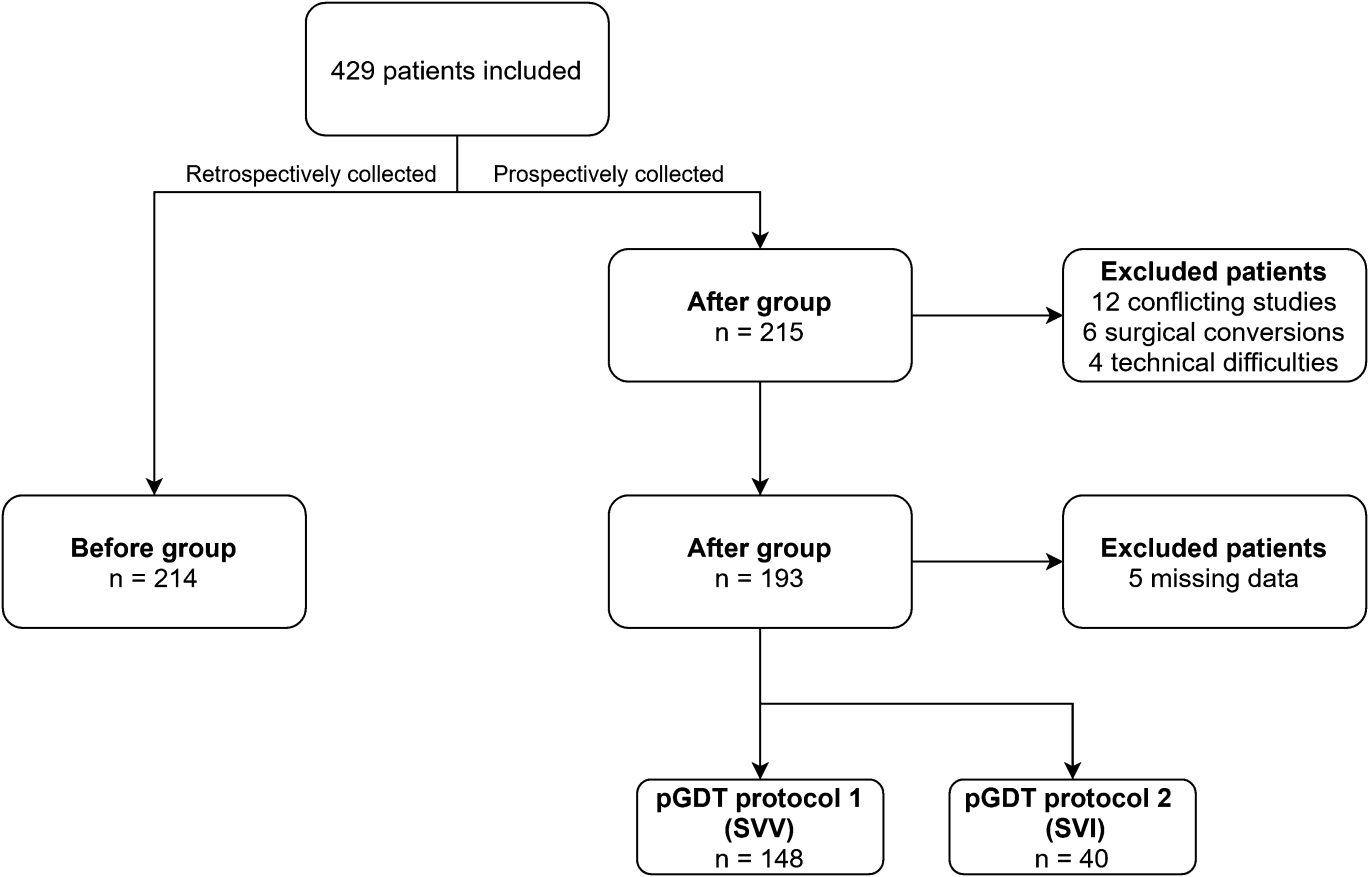
Flowchart of the inclusion of patients

In total, 429 patients were eligible for this study, of which 214 were included in the before-group and 215 were screened for the after-group. In the after-group, 22 patients were excluded due to participation in conflicting interventional studies, intraoperative conversion from curative to palliative care, or technical difficulties (Fig. 2). Finally, 193 patients in the after-group were included and suitable for analysis, of which 188 patients had sufficient data for protocol compliance analysis. 153 patients (79%) were treated according to the SVV-based pGDT treatment algorithm (pGDT 1), of which in 5 patients hemodynamic data could not be retrieved. These patients were excluded from the protocol compliance analysis. Forty patients (21%) were treated according to the SVI-based pGDT treatment algorithm (pGDT 2).

No relevant differences existed in patient characteristics between the before-group and the after-group (Table 1).

**Table 1:** Characteristics and perioperative data of patients included before and after implementation of a perioperative goal-directed therapy protocol. Values are mean (SD), median [IQR] or number (proportion)

		Before group (n = 214)	After group (n = 193)	p value
Age (years)		66 (10.7)	66 (11.1)	0.66
Sex (male)		138 (64%)	122 (63%)	0.79
BMI (kg m^− 2^)		26.4 (5.4)	26.5 (4.4)	0.77
ASA classification	ASA 1	11 (5%)	21 (11%)	0.017
	ASA 2	116 (54%)	98 (50%)	
	ASA 3	78 (36%)	73 (38%)	
	ASA 4	9 (4%)	1 (1%)	
Included types of surgery	PPPD	48 (22%)	48 (25%)	0.97
	APR	62 (29%)	57 (30%)	
	Open AAA	31 (15%)	25 (13%)	
	Esophagus resection	25 (12%)	22 (11%)	
	Femoral-popliteal bypass surgery	48 (22%)	41 (21%)	
Procedure duration; min		300 [210–450]	336 [242–469]	0.046
Blood loss; mL		500 [150–1050]	700 [250–1300]	0.026
Urine production; mL		520 [300–900]	680 [350–1000]	0.047
Crystalloids used		200	183	1.000
Crystalloid rate (mL kg^− 1^ min^− 1^)		0.17 [0.12–0.24]	0.17 [0.13–0.22]	0.64
Colloids used		111	109	0.42
Colloid rate (mL kg^− 1^ min^− 1^)		0.03 [0.02–0.06]	0.03 [0.02–0.04]	0.021
Packed RBC used		40	42	0.48
Packed RBC (mL kg^− 1^ min^− 1^)		0.03 [0.02–0.04]	0.02 [0.01–0.04]	0.17
Norepinephrine used		148	181	< 0.001*
Norepinephrine dose (µg kg^− 1^ min^− 1^)		0.08 [0.04–0.13]	0.06 [0.03–0.1]	0.009
Phenylephrine used		66 (31)	30 (16)	< 0.001*
Dobutamine used		5 (2)	30 (16)	< 0.001*
Dobutamine dose (µg kg^− 1^ min^− 1^)		3.11 [0.17–8.28]	2.35 [1.28–3.88]	1.000

Given is the number or number (percentage)

*BMI *body mass index, *ASA* American Society of Anesthesiology, *PPPD *pylorus-preserving pancreaticoduodenectomy, *APR* abdominoperineal resection, *AAA* abdominal aorta aneurysm, *RBC *red blood cells

*Bonferroni corrected p values of 0.0025 or lower are deemed significant

### **Intraoperative hemodynamic management**

No differences between both groups with respect to the use of crystalloids were observed (Table 1). Although colloids were used in a comparable number of patients, the mean dosage of colloids was higher in the before-group. Norepinephrine was used less frequently in the before-group compared to the after-group (148 vs. 181 patients; 69% vs. 94%; p < 0.001), while the contrary was true for phenylephrine: (66 vs. 30 patients; 31% vs. 16%; p < 0.001). However, no difference in the mean administered dosages of norepinephrine and phenylephrine between both groups was observed (Table 1).

### **Postoperative complications**

Expectedly, implementation of the pGDT protocol was associated with a reduction in the total sum of complications (n = 414 in the before group vs. n = 282 in the after group, p = 0.031; Table 2, meaning that the complication rate per patient, decreased from 1.93 complications per patient in the before-group to 1.46 complications per patient in the after-group. The cumulative complication score was also lower in the after-group compared to the before-group (3.0 vs. 4.4 respectively; p = 0.009). Stratifying complications based on severity showed no difference for mild or moderate complications between the before and after-group (p = 0.311 and p = 0.177, respectively) (Table 2). However, the number of severe complications was reduced in the after-group compared to the before-group (n = 95 vs. n = 47 respectively, p = 0.003), meaning that the complication rate per patient reduced from 0.44 to 0.24. The number of severe complications requiring an invasive procedure without general anesthesia was also reduced, from 37 to 14 respectively (p = 0.006; rate per patient 0.17 (before-group) to 0.07 (after group). However, the occurrence of organ failure and postoperative death did not differ between groups, which was also true for the number of severe complications requiring a re-intervention under general anesthesia (Table 2).

**Table 2:** The number of postoperative complications in patients undergoing surgery before and after implementation of a perioperative goal-directed therapy protocol

		Before group (n = 214)	After group (n = 193)	p value
Patients with complications		154 (72%)	124 (64%)	0.10
Mild/moderate complications	Mild	119	95	0.31
	Moderate	188	133	0.18
	Total complications	307	228	0.097
Severe complications	Invasive procedure/no GA*	37	14	0.006*
	Invasive procedure/GA	43	25	0.07
	Organ failure	15	8	0.16
	Total complications	95	47	0.003*
(Postoperative) death		12 (6%)	7 (4%)	0.34
Total complications	Sum of complications	414	282	0.031*
(including death)	Cumulative complication score	4.4	3.0	0.009*

Data are given as number or as number (percentage)

*GA *general anesthesia

*p < 0.05

### **Protocol compliance**

**Fig. 3:**
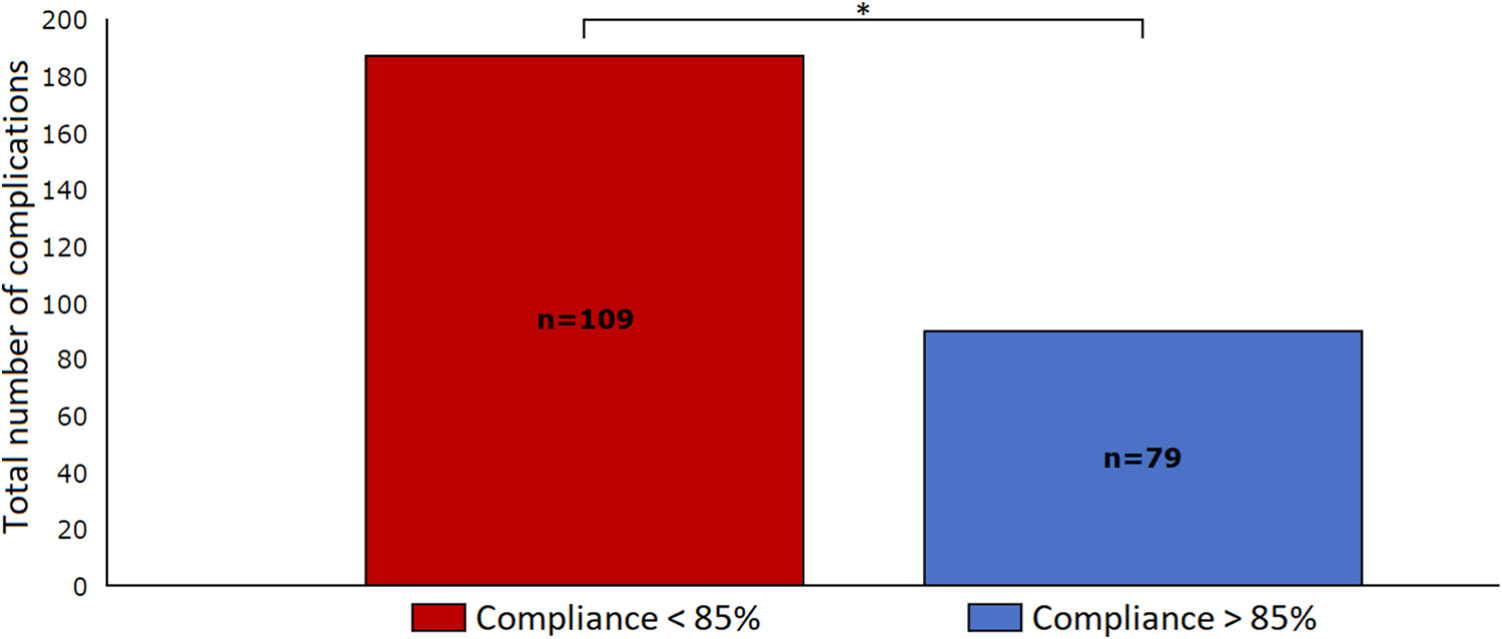
The association between protocol compliance based on SVV and SVI, and the total number of postoperative complications. * p = 0.01

Of the procedures in which pGDT was used, 79 procedures (42%) reached high protocol compliance for SVV or SVI (Table 3), while only 39 (21%) of the 188 procedures reached high protocol compliance for the CI target (Table 4). Procedures with a high compliance for the either the SVV or SVI target were associated with a lower occurrence of postoperative complications compared to procedures with low protocol compliance (OR 0.45; 95% CI 0.25–0.84). High SVV or SVI compliance was also associated with a reduction in mild/moderate (n = 149 vs. n = 74; p = 0.023) and severe complications (n = 34 vs. n = 13; p = 0.015) compared to low protocol compliance. In addition, the cumulative complications score was reduced in procedures with high compliance (3.54 vs. 2.29; p = 0.005) (Table 3). The total number of complications was reduced by 34% in procedures with high SVV or SVI compliance (90 vs. 187; p = 0.01) (Fig. 3). No differences were found for postoperative death between high and low protocol compliance for SVV or SVI. No differences in the occurrence or number of complications or postoperative death were observed between high and low compliance for the CI target.

**Table 3:** The number of postoperative complications in patients undergoing surgery based on compliance with stroke volume variation/stroke volume index

	Low compliance with SVV or SVI (n = 109)	High compliance with SVV or SVI (n = 79)	P-value
Patients with complications	79 (72%)	43 (54%)	0.011*
Mild/moderate complications	149	74	0.023*
Severe complications	34	13	0.015*
Total number of complications	187	90	0.01*
Cumulative complication score	3.54	2.29	0.005*
60-day death postoperatively	4 (4%)	3 (4%)	0.964

Data is given as number or number (percentage)

*SVV* stroke volume variation, *SVI* stroke volume index

*p-values of 0.05 or lower are deemed significant

## **Discussion**

In this before-and-after study, implementation of a pGDT protocol was associated with a reduction in postoperative complications in patients undergoing HRS. Importantly, high compliance with the SVV and SVI section of the pGDT protocol was associated with a further reduction in mild, moderate and severe postoperative complications. Our study shows that in order to improve postoperative outcomes after the implementation of pGDT protocols, it is relevant to assess and increase protocol compliance of the attending caregivers.

**Table 4:** The number of complications in patients undergoing surgery based on compliance with cardiac index

	Low compliance with CI (n = 149)	High compliance with CI (n = 39)	P-value
Patients with complications	99 (66%)	23 (59%)	0.39
Mild/moderate complications	181	42	0.41
Severe complications	39	8	0.78
Total number of complications	226	51	0.48
Cumulative complication score	3.1	2.6	0.39
60-day death postoperatively	6 (4%)	1 (3%)	0.67

Data is given as numbers or as number (percentage)

*CI* cardiac index

### **Reduction of postoperative complications**

It was not unexpected that the implementation of our pGDT protocol was associated with a reduction in the incidence of postoperative complications. The observation of an improvement in postoperative patient outcomes following pGDT protocol implementation is in accordance with those of several systematic reviews with meta-analysis [2, 26]. In these systematic reviews, most often gastrointestinal surgery was assessed. We restricted our analysis to five surgical procedures that are performed frequently in our institution. Furthermore, to the best of our knowledge, there were no concurrent quality improvement projects for patients undergoing these procedures.

While many studies have shown that pGDT implementation results in lower postoperative morbidity [15, 27–29], this is not per se self-evident. In one study with similarities to our study, i.e., a quality improvement program to evaluate a pGDT protocol in esophageal surgical procedures, no reduction was found regarding overall morbidity, length of hospital stay, or mortality [16]. However, the authors of that study mainly included patients undergoing minimally invasive laparoscopic esophagectomies, which are associated with fewer postoperative complications compared to open transthoracic esophagectomies [30]. In our study, only open esophagectomies were included, which may explain the conflicting results regarding the influence of pGDT on postoperative complications.

Regarding vasoactive agents, norepinephrine and dobutamine were used more frequently in the after-group, while usage of phenylephrine was reduced. The increased dobutamine usage may be due to our protocols recommending dobutamine to increase CI if patients are no longer fluid responsive.

### **Influence of protocol compliance on complication incidence**

The primary aim of our study was to assess whether compliance with a pGDT protocol was associated with postoperative outcomes. We found that high compliance (i.e., > 85% of the time) for the SVV and SVI-based algorithm was associated with a lower overall complication rate, with a lower number of mild, moderate, and severe complications. Interestingly though, no statistically significant differences in complication rate or postoperative death between the procedures with high and low compliance for the CI target were found, probably due to a low percentage of patients in which the CI targets were achieved.

Our results show that in only 46% of procedures, high protocol compliance with SVV/SVI was achieved and that this was true in only 24% of the patients regarding the CI targets. Multiple explanations exist for these rather low protocol compliance rates. Regarding SVV/SVI, low protocol compliance could be a consequence of hemodynamic instability during the operation, e.g. by blood loss. Since a fluid challenge is performed in 10 minutes and may need to be repeated before SVV or SVI is within their respective targets, protocol compliance could drop below the 85% cut-off point while still performing the protocol correctly. Also, attention to our protocol could be hampered by, for example, other intraoperative events requiring intervention. Additionally, caregivers may have based their hemodynamic management for individual patients on non-algorithm based individual preferences.

The even lower compliance with the CI target (only 24% of patients achieved the target CI) could be explained by the fact that the pGDT algorithm prioritizes SVV (pGDT 1) or SVI (pGDT 2) over CI. Both treatment algorithms recommend targeting and improving SVV or SVI first, and considering dobutamine only after SVV or SVI was optimized or when the patient was considered fluid unresponsive. The more “secondary” place of CI in these treatment algorithms could have resulted in CI being considered less important compared to SVV or SVI—and it may have resulted in a delay in optimizing CI. Another reason might be that our anesthesiologists were not comfortable with administering dobutamine to increase CI in patients undergoing abdominal surgery, or they could have been afraid of side effects such as tachycardia and arrhythmias. Although dobutamine was used more frequently in the after-group, it was still only used in a minority (16%) of patients. In addition, it must be realized that although SVI and CI are more physiologically linked than SVV and SVI are, we bundled the compliance analysis for SVV and SVI on one hand, and CI on the other hand, since it was our aim to predominantly assess the compliance of caregivers to both treatment algorithms irrespective of the exact hemodynamic variables that were used.

### **Implications and generalizability**

Before-after studies show the effects of implementing a protocol more realistically than a randomized controlled trial. Nevertheless, this type of research has the disadvantage that confounders potentially influence results [19]. Protocol compliance as confounder has been studied in other areas of perioperative and postoperative research, which have shown that higher protocol compliance leads to better patient outcomes [9, 31, 32]. However, few studies have investigated the influence that protocol compliance could have on the occurrence of postoperative complications [33]. An additional disadvantage of before-after evaluation is the difficulty to relate interventions to the effects on a patient’s perioperative physiological response. Using intervention-based protocol compliance could enable us to record a patient’s response to a specific intervention and determine what causes lower protocol compliance. Therefore, the results of our study could be considered a first impression of the influence of pGDT protocol compliance on patient outcomes.

### **Limitations**

Our study has several limitations. First, as we intentionally selected a limited number of HRS procedures, our findings cannot be generalized to other interventions. Second, our pGDT protocol was introduced as an addition to current standard practice for anesthesiologists and was not prioritized over other institutional protocols used during the procedure. While this may have affected pGDT protocol compliance, it does provide a more realistic view of a perioperative setting in which multiple protocols are being used simultaneously. Third, our pGDT protocol focused mainly on the intraoperative period. Hemodynamic changes and their treatment in the early postoperative period (ICU or post-anesthetic care unit) could have modified the outcome effects of our hemodynamic optimization efforts. Of note, the low compliance rate for CI that we observed, may “mask” a statistical association with postoperative outcome (i.e. a type II error), and a larger number of patients in whom CI compliance was sufficiently high may have given different results.

Finally, it cannot be excluded that a higher compliance was more easily reached in more “stable” patients, although this was not reflected by relevant data (e.g. there were no differences between low and high compliance with respect to ASA classification, surgery duration, or blood loss).

In summary, our study shows that if quality improvement is intended by the implementation of perioperative goal-directed therapy in patients undergoing high-risk surgery, it is of particular importance to monitor and achieve a high protocol compliance in order to fully optimize postoperative outcomes.

## Electronic supplementary material

Below is the link to the electronic supplementary material.

Electronic supplementary material 1 (PDF 239 kb)—pGDT protocol 2. SVI = Stroke volume index. CI = Cardiac index. Both variables were obtained by using the EV1000monitoring system, utilizing uncalibrated pulse-wave contour analysis.
