# Supplementary material for: The effect of compliance with a perioperative goal-directed therapy protocol on outcomes after high-risk surgery: a before-after study
Source: J Clin Monit Comput. 2020 Sep 12;35(5):1193–202. doi: 10.1007/s10877-020-00585-w (PMC7486974; doi:10.1007/s10877-020-00585-w)
Supplement: Supplementary file 1 — Electronic supplementary material 1 (PDF 239 kb)—pGDT protocol 2. SVI = Stroke volume index. CI = Cardiac index. Both variables were obtained by using the EV1000monitoring system, utilizing uncalibrated pulse-wave contour analysis. [file 10877_2020_585_MOESM1_ESM.pdf]

Electronic Supplementary Material: pGDT protocol 2

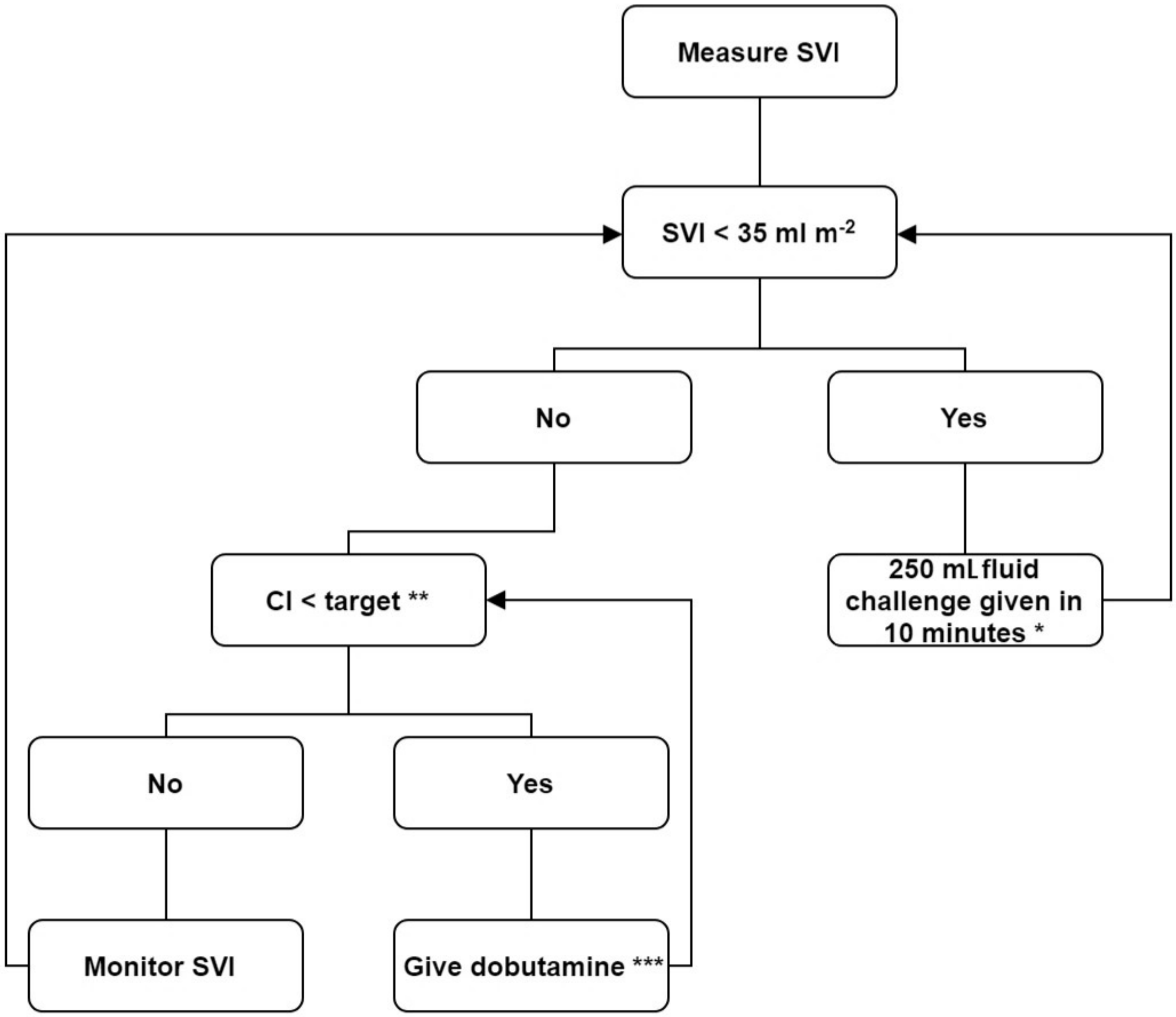

pGDT protocol 2

- * Fluid challenge: colloids (max. 4 units) or crystalloids (e.g. ringer's lactate)
  Alternative option: transfuse 1 unit of red blood cells (only when combined with low Hb levels)
- ** CI target is age-dependent
  - < 60 years: 2.8 L min^{-1} m^{-2}
  - 60 – 75 years: 2.6 L min^{-1} m^{-2}
  - >75 years: 2.4 L min^{-1} m^{-2}
- *** Starting dosage: 2.5 µg kg^{-1} min^{-1}
  Alternative option: noradrenaline (syringes of 5mg/50mL or 10mg/50mL), starting dosage 0.02 – 0.04 µg kg^{-1} min^{-1}
  When a central venous catheter is absent, consider:
  Ephedrine / phenylephrine
  Noradrenaline (1mg/50mL)

Other hemodynamic targets

- Standard fluid intake: 2mL kg^{-1} h^{-1} crystalloids
- Hb targets according to hospital guidelines for adults
- Heart frequency < 100 beats/minute
